# Predicting measurement continuity in home blood pressure monitoring using machine learning

**DOI:** 10.1038/s41440-025-02444-0

**Published:** 2025-11-07

**Authors:** Asami Matsumoto, Yohei Mineharu, Hirohiko Kohjitani, Hiroshi Koshimizu, Yasushi Okuno

**Affiliations:** 1 Department of Biomedical Data Intelligence, Kyoto University Graduate School of Medicine, Kyoto, Japan; 2 Omron Healthcare Co., Ltd., Kyoto, Japan; 3 Department of Artificial Intelligence in Healthcare and Medicine, Kyoto University Graduate School of Medicine, Kyoto, Japan

**Keywords:** Digital hypertension, Implemental hypertension, Morning hypertension, Patient compliance, Telemedicine

## Abstract

Hypertension is a leading risk factor for cardiovascular diseases, thereby necessitating effective management through regular blood pressure monitoring. Although home monitoring is beneficial for managing hypertension, maintaining consistent measurement frequency remains challenging. This study aimed to develop a model to predict measurement inactivity and to identify clinically relevant risk factors for declining adherence using machine learning, thereby allowing for targeted interventions. Using a large-scale dataset (>199 million measurement records) from 295,758 health app users, we employed a LightGBM (Light Gradient Boosting Machine) model to predict future inactivity according to 2-week measurement patterns and users’ demographics. The model demonstrated high predictive accuracy, with areas under the receiver operating characteristic curve of 0.930 and 0.851 for 28- and 56-day predictions, respectively. SHAP (SHapley Additive exPlanations) analysis revealed elevated dropout risks among both younger and older participants, women, and users who did not report sex information. The maximum systolic blood pressure (SBP) recorded during the 2-week period was also identified as a significant predictor of dropout, showing a U-shaped association wherein both low and high extremes increased the risk. This maximum SBP value, which is rarely used in routine clinical assessments, offered unique insights into dropout behavior, further supported by descriptive statistics. Additionally, a reduction in weekday measurement frequency showed to be a major predictor of future discontinuation. Therefore, our model can identify dropout factors that are difficult to detect by conventional methods, and through accurate prediction, it supports early clinical interventions to improve monitoring adherence and blood pressure control.

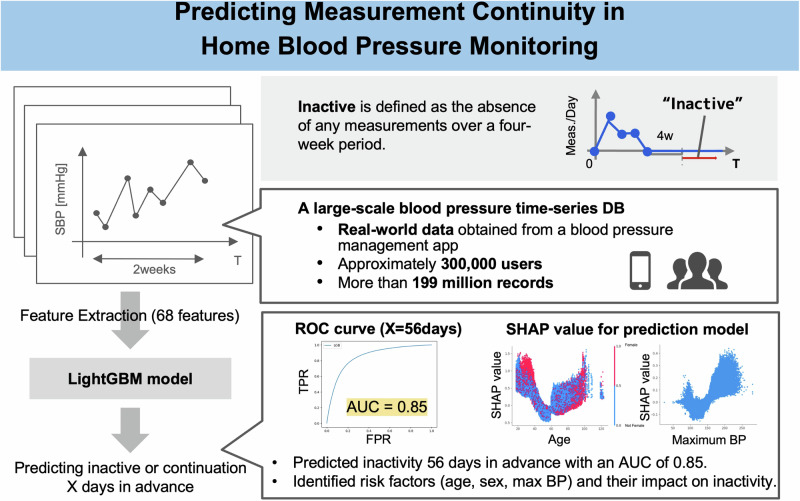

## Introduction

Hypertension is a significant risk factor for cardiovascular diseases. According to a World Health Organization survey, approximately one-third of adults aged 30–79 years have hypertension. Among them, 23% of women and 18% of men have uncontrolled blood pressure [1]. Thus, effective blood pressure management is essential for preventing and treating cardiovascular diseases.

Self-monitoring of blood pressure at home has been reported to be effective in managing hypertension [2–4], suggesting that regular home blood pressure monitoring significantly contributes to reducing blood pressure in patients with chronic hypertension. Therefore, it plays a crucial role in hypertensive management. Existing hypertension treatment guidelines also emphasize the importance of regular home blood pressure measurements. For instance, the American hypertension treatment guidelines recommend blood pressure measurement twice daily [5], particularly after any treatment changes or before medical consultations. However, maintaining vital sign measurements at a high frequency is challenging for many patients. Digital health services targeting chronic conditions report high discontinuation rates, exceeding 75% [6], indicating the challenges of sustained engagement [6–8]. Furthermore, lower measurement frequency correlates with reduced treatment efficacy, suggesting that adherence to and retention in home blood pressure monitoring are crucial for effective blood pressure management [9, 10].

Considering this context, healthcare professionals are increasingly needed to intervene to ensure sustained measurements. However, the economic and time-related costs associated with such interventions remain to be significant challenges. Constant feedback and reminders from healthcare professionals can be a substantial burden, considering their limited daily time. Therefore, a selective intervention approach that targets patients at risk of measurement discontinuation is necessary to enhance efficiency. Several studies have proposed methods for predicting measurement engagement according to the usage patterns of health management applications. Serrano et al. [11] and Lee et al. [12] found correlations between specific application usage features and sustained user engagement. Additionally, a model has been developed to predict dropouts after 4 weeks, mainly using information such as intervention status in a specific health management platform and the timing of measurements [13]. However, the relationship between time-series vital sign data and measurement engagement remains unclear.

Pratap et al. analyzed data from more than 100,000 individuals to investigate the factors that influence the continuation of remote digital health services [14]. This comprehensive analysis included multiple studies; however, a specific dropout prediction model was not developed, and the data backgrounds were heterogeneous.

This study aimed to develop a model to predict measurement inactivity among users of home blood pressure monitors using time-series vital sign data and to identify clinically relevant risk factors for declining adherence using machine learning, thereby allowing for targeted interventions. Given the large-scale, which included over 199 million records from 295,758 users, multivariate nature of this dataset, we employed a Light Gradient Boosting Machine (LightGBM) [15] model for prediction and SHapley Additive exPlanations (SHAP) [16] for interpretation. This prediction model will enable timely development of effective interventions before the patients completely stop performing measurements, thereby improving patient adherence and retention in the measurements. It is also expected to efficiently and uniformly support tasks that traditionally relied on manual intervention.

## Methods

### Dataset

The Institutional Review Boards of Omron Healthcare Co., Ltd and Kyoto University approved this study (approval numbers: Omron Healthcare Co., Ltd “IRB-2323(3)” and Kyoto University “R4222”). Users of the Omron Connect application provided by Omron Healthcare Co., Ltd were informed about the purpose of data use through the terms of service; additionally, downloading the application and registering a new account indicated that they provided consent [17].

This study used data collected from Japanese users through Omron Connect from the start of data accumulation in November 2016 to April 2023. The inclusion criteria for study participants were individuals who were 18 years old and above based on the mandatory birth date field and who owned an Omron upper-arm blood pressure monitor connected to the Omron Connect application. Omron Connect accumulated data from upper-arm blood pressure monitors manufactured by Omron Healthcare. These data were then extracted and analyzed. Such data included systolic and diastolic blood pressure (SBP and DBP, respectively) readings and pulse rate measured simultaneously, measurement dates, and the country where the device was registered, as well as sex and birth date (year and month only) reported by the users during service registration.

### Definition of “inactive”

**Fig. 1:**
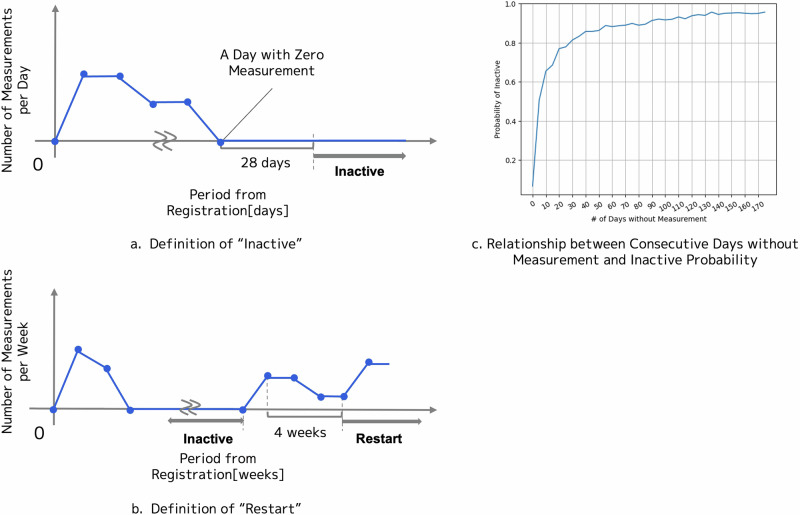
**a** Diagram showing the definition of “inactive.” The *x*-axis represents the number of elapsed days, whereas the *y*-axis represents the number of measurements per day. **b** Diagram showing the definition of “resuming” measurements. The *x*-axis represents the number of elapsed weeks, whereas the *y*-axis represents the number of measurements per week. **c** Relationship between consecutive days without measurements and the probability of not resuming measurements within 6 months. The *x*-axis represents the number of consecutive days without measurements, whereas the *y*-axis represents the probability of not resuming measurements within 6 months. Users who did not measure for 28 days had an 80% chance of not resuming measurements

In this study, “inactive” was defined as the absence of any measurements for 4 weeks (28 days) (Fig. 1a). This definition was based on the following points. We analyzed the relationship between consecutive days without measurements and the probability of not resuming measurements within 6 months. Statistical analysis revealed that ~80% of users who did not perform measurements for 28 days did not resume measurements for at least 180 days (Fig. 1c). Measuring at least once a week for 4 consecutive weeks defined resuming measurements (Fig. 1b). This distinction helps differentiate between sporadic and continuous measurements. Additionally, the definition of 28 days (4 weeks) without measurements is consistent with that used in previous studies.

### Analysis approach

**Fig. 2:**
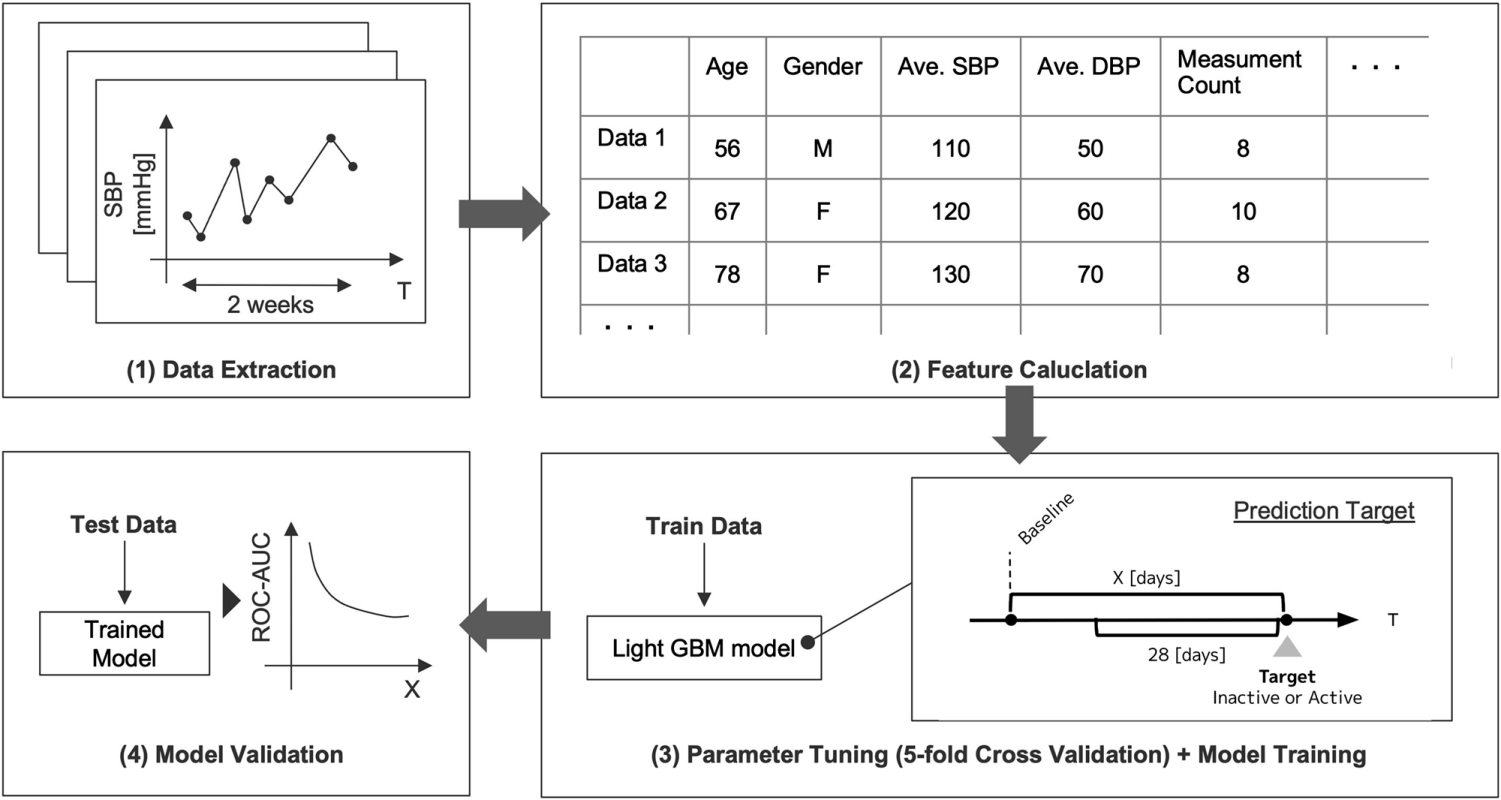
Data preprocessing and model construction

To predict “inactive” status from the measurement data obtained from the dataset, we segmented the input measurement data into 14-day periods and calculated the features representing the measurement patterns.(Fig. 2) The data of each user were divided into multiple nonoverlapping segments. At least 1 week of data input is needed to capture measurement patterns. Although longer periods improve prediction accuracy, overextending the period deviates from the objective of early prediction of inactive states. Therefore, we considered 14 days optimal.

We calculated 68 features representing the measurement patterns (Supplementary Table 1). Combined with user attributes (age and sex), 65 features were used as inputs to the model. Regarding sex-related features, two flags were defined to indicate whether the user is a female and whether sex information was provided. The prediction target was the inactive status from 28 to 91 days later in 7-day increments (denoted as variable X). The length of this period balances the potential to prevent measurement dropout with the prediction accuracy. The changes within this period are presented in the Results section.

To predict measurement inactivity based on these features, we developed models using LightGBM and logistic regression. Clarifying the basis for the model’s decisions is necessary to integrate the model into a remote patient monitoring (RPM) service; therefore, we selected these two commonly used models for their high interpretability. LightGBM was particularly suited for this study because it can process massive datasets more quickly and with lower memory requirements than other tree-based techniques, such as Random Forests [18] and XGBoost [19], without sacrificing predictive performance.

During model training, we conducted fivefold cross-validation and used Optuna’s Bayesian optimization algorithm for parameter tuning. Using the selected parameters, we calculated the area under the receiver operating characteristic curve (ROC-AUC) for the test data and then compared the accuracy of the LightGBM and logistic regression models.

In addition, we also employed SHAP to interpret model predictions. SHAP quantifies the contribution of each input feature by assigning values that reflect both the magnitude and the direction of its impact on the model’s output. In other words, SHAP values reveal which factors, such as measurement frequency or time since the last measurement, most strongly influence the predicted risk of dropout. Unlike descriptive statistics, which merely summarize data using measures such as means and medians, SHAP analysis provides feature-specific attributions that indicate not only the relative importance of each factor but also whether a feature increases or decreases dropout risk, and to what extent.

### Model validation strategy

A push notification feature was implemented on February 26, 2018, during our data collection period. Therefore, we first conducted a confirmatory analysis to ensure that the model’s predictive accuracy was not affected by the presence of this feature in the training data. This analysis confirmed that accuracy was consistent regardless of the training data period (for the detailed methodology and results, see Supplementary Figs. 1 and 2).

Based on this confirmation, we employed a data-splitting strategy for our primary evaluation designed to maximize the available training data (Supplementary Fig. 3). In this approach, all users from the period without push notifications were designated as “Test 1” to evaluate generalization performance. An equivalent number of users were randomly selected from the period with push notifications to form a comparable dataset, defined as “Test 2.” All remaining user data not included in Test 1 or Test 2 were used for training the final model.

## Results

### User statistics

This dataset includes blood pressure measurement data from 295,758 users (Table 1). The mean age of the users was 55.5 ± 11.4 years. Regarding sex distribution, 61.4% were male, 29.1% were female, and 9.5% were those who did not specify their sex. The median measurement period (the number of days from the first measurement to the last measurement) was 370 days, with an interquartile range (Q3–Q1) of 765 days. During this period, the median number of measurements was 229, with an interquartile range of 708 measurements. In addition, the median SBP was 127.8 mmHg, with an interquartile range of 16.1 mmHg.

**Table 1:** Characteristics of users

Number of users, *N*	295,758
Age, mean (SD)	55.5 (11.4)
Male, *N* (%)	181,528 (61.4)
Female, *N* (%)	86,069 (29.1)
Sex not provided, *N* (%)	28,161 (9.5)
Measurement period (days from the first measurement to the last measurement) [days], median (Q1, Q3)	370 (87, 852)
Number of measurements during the measurement period, median (Q1, Q3)	229 (56, 764)
Average systolic blood pressure [mmHg], median (Q1, Q3)	127.8 (119.7, 135.8)

### Evaluation outcomes

We evaluated the model’s performance using the primary data-splitting strategy detailed in the Methods section (Supplementary Fig. 3).

**Fig. 3:**
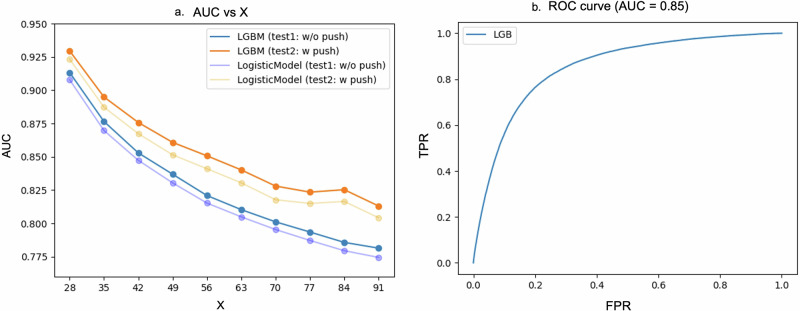
**a** Graph showing the relationship between *X* (the number of days ahead to predict) and the prediction accuracy of the LightGBM and logistic regression models. The *x*-axis represents *X*, whereas the *y*-axis represents the area under the receiver operating characteristic curve (ROC-AUC). The orange and blue curves represent Test 1 and Test 2 evaluations for the LightGBM model, whereas the yellow and light blue lines represent those for the logistic regression model, respectively. **b** Graph depicting the ROC curve of the LightGBM model that predicted the measurement dropout 56 days in advance. The *x*-axis and *y*-axis represent the false positive rate (FPR) and true positive rate (TPR), respectively

Figure 3a presents the changes in the ROC-AUC for the LightGBM and logistic regression models as the prediction horizon (*X*) varied from 28 to 91 days in 7-day increments. As X increased, the prediction task became increasingly challenging, exponentially decreasing the accuracy. When using test data from the push notification period, the ROC-AUC of the LightGBM model was 0.930 for *X* = 28 and 0.851 for *X* = 56. Given that LightGBM outperformed logistic regression in terms of accuracy across all values of *X*, subsequent discussions on SHAP values and accuracy focused on the LightGBM model.

**Fig. 4:**
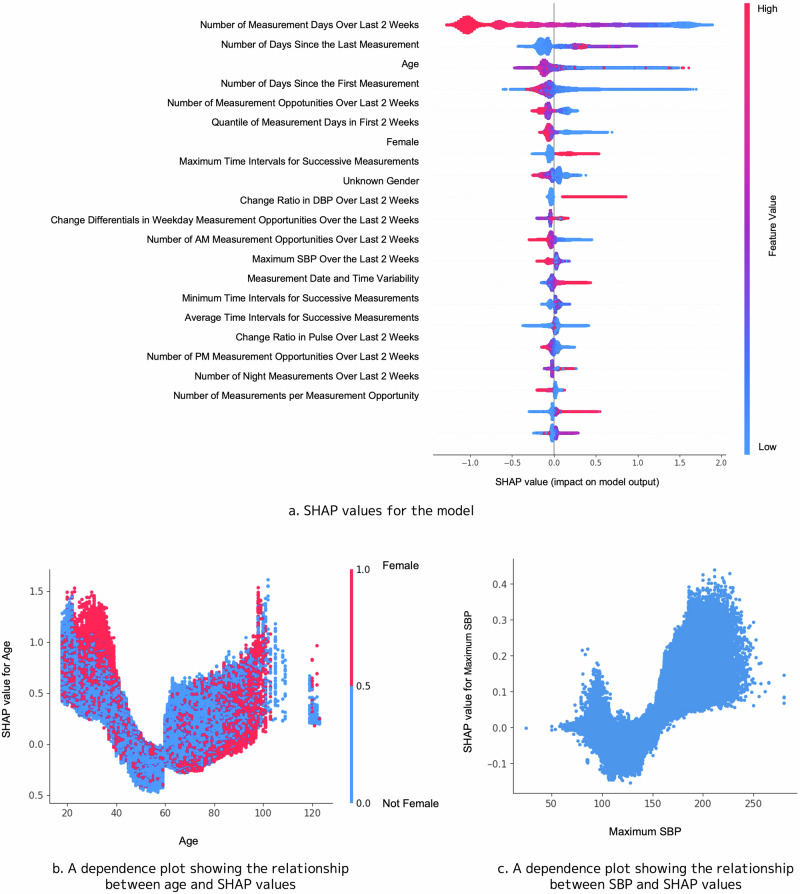
**a** Graph illustrating the SHAP values for the model predicting the measurement dropout 56 days in advance. The *x*-axis represents the SHAP values, and the color of the plotted points indicates the magnitude of each feature’s value. **b** Dependence plot showing the relationship between age and the SHAP values. The *x*-axis represents age, whereas the *y*-axis represents the SHAP values. The red and blue points represent females and nonfemales, respectively. **c** Dependence plot showing the relationship between the maximum SBP and SHAP values. The *x*-axis and *y*-axis represent the maximum SBP and SHAP values, respectively

Figure 4a presents the SHAP values when *X* was set to 56. The number of days measured within a 2-week period was the most significant contributor to dropout. The probability of dropout tended to increase as the number of days since the last measurement increased. Among other measurement pattern–related features, the shorter number of days since the first measurement, the fewer measurements in the first 2 weeks after measurement initiation, and the greater variation in measurement frequency ranked highly. Regarding demographic attributes, age and sex were among the top-ranked features. Figure 4b reveals that dropout tendencies are higher among younger individuals aged around 30 years and those aged ≥60 years. However, when examining the dependence plot for age according to sex, younger women had a significantly higher tendency to drop out than men. Furthermore, we observed a pattern wherein those who did not specify their sex in the application had a higher dropout probability than those who provided sex information. Regarding blood pressure–related features, the dependence plot for maximum SBP revealed a U-shaped association with dropout risk (Figure 4c). This plot showed that an increased tendency to drop out at both relatively low and high SBP values, with the risk escalating sharply above the threshold of ~150 mmHg on the higher end. This U-shaped pattern was consistently observed across all sex groups—female, male, and those who did not report their sex—as shown in Supplementary Fig. 4.

Changes in measurement opportunities, calculated as the difference between the first and second weeks, also emerged as a significant factor associated with dropout.

Statistical analysis confirmed the overall relevance of this metric, demonstrating that these changes significantly differed between the continuation and dropout groups across all time frames: the entire week, weekdays, and weekends (as detailed in Supplementary Fig. 5). Notably, SHAP analysis further emphasized the importance of weekday-specific patterns within this metric, with the feature representing weekday changes ranking within the top 15 predictors.

### Additional descriptive analysis

**Fig. 5:**
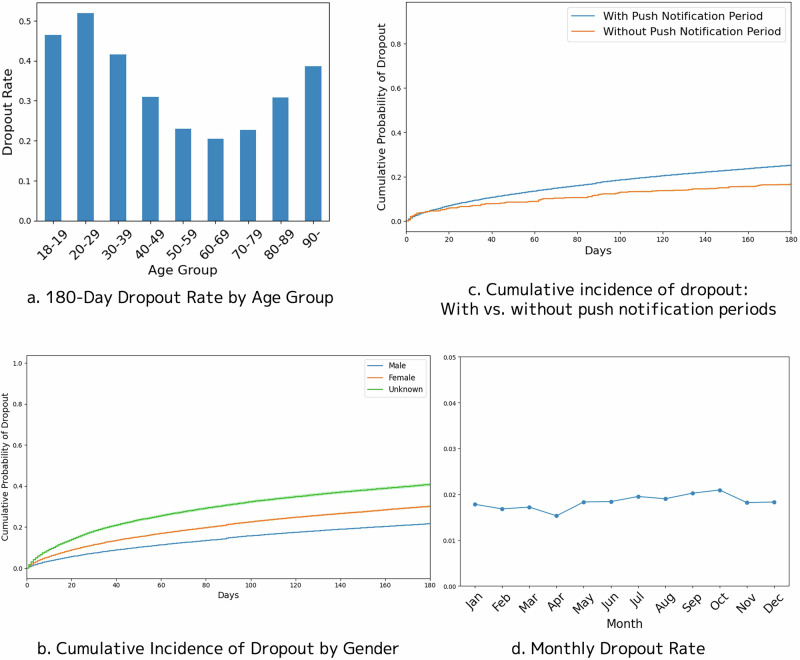
**a** Graph showing 180-day dropout rates by age group. The *x*-axis represents age categories, whereas the *y*-axis represents the proportion of users who discontinued measurements within 180 days. **b** Cumulative incidence functions stratified by sex. The *x*-axis represents the number of days since the first measurement, whereas the *y*-axis represents the cumulative probability of dropout, with separate curves for females, males, and users of unspecified sex. **c** Comparison of cumulative incidence functions between the push notification period and the non–push notification period. The *x*-axis represents the number of days since the first measurement, whereas the *y*-axis represents the cumulative probability of dropout for each period. **d** Monthly dropout rate plot illustrating seasonal variation. The *x*-axis represents the calendar month, whereas the *y*-axis represents the percentage of users who discontinued measurements within each month

We conducted additional analyses to evaluate dropout risk in relation to both demographic factors and initial blood pressure measurements. This aimed to verify consistency between the underlying data distribution and the feature importance rankings indicated by SHAP values in the machine learning model. When plotted by age, the dropout rates were higher among users aged 40 years and younger, decreased among those in their 50s to 70s, and increased again among users aged 80 years and older, showing a U-shaped trend (Fig. 5a). We then estimated the cumulative incidence function stratified by sex; as observed, females had a higher cumulative probability of dropout than males, with the highest incidence among users who did not specify their sex (Fig. 5b).

The cumulative incidence functions for users whose measurements were taken during the push notification period were further compared with those during the non–push notification period. Of note, the push notification period refers to the time after the push notification feature was implemented, implying that while users in this period could receive notifications, not all necessarily did so. To ensure comparability between these two groups, we made adjustments for age and sex. The cumulative incidence of dropout was lower in the non–push notification period than in the push notification period (Fig. 5c).

Finally, to investigate the potential influence of seasonality on dropout risk, we calculated and plotted monthly dropout rates. Given the possible impact of the COVID-19 pandemic, we specifically analyzed data from 1 year before and 1 year after January 2020, when the first COVID-19 case was confirmed in Japan. This analysis did not reveal any marked seasonal fluctuations in dropout rates (Fig. 5d).

## Discussion

### Principal results

This study demonstrated that future dropout in measurement can be predicted with high accuracy according to a 2-week measurement pattern using a large dataset of blood pressure measurement records from ~300,000 users.

In particular, we achieved AUC values of ~0.930 and 0.851 for predicting dropout at 28 and 56 days. The ability to predict dropout 4 weeks in advance using only 2 weeks of data is significant. According to Wu et al. [20], the effectiveness of interventions can be predicted on the basis of 4 weeks of measurement patterns; thus, by predicting dropout within a 2-week period and improving continuity through intervention, treatment efficacy may be enhanced. The effects of interventions on prediction accuracy are crucial. Our data included periods with and without push notifications, allowing us to evaluate the impact of interventions. Ultimately, we found that the presence or absence of interventions did not affect prediction accuracy, demonstrating that our model is universal and robust. Regarding dropout incidence, results suggest that the dropout rate was slightly lower in the non–push notification period than in the push notification period. These differences, however, may arise from calendar-time effects, including shifts in user demographics, seasonal variations, application design updates, and promotional activities, rather than the notifications themselves. Given that this retrospective analysis lies outside the primary objectives of our predictive modeling study, we have treated it as exploratory and did not further investigate into causal mechanisms.

The SHAP analysis revealed that the risk of measurement dropout increased when the users had fewer measurement days, a shorter duration from the initial measurement, fewer measurements in the first 2 weeks, and relatively low or high maximum SBP values recorded during that period. In particular, a maximum SBP exceeding 150 mmHg markedly elevated the likelihood of dropout. This finding suggests that even a single high SBP reading may negatively impact user motivation. Exposure to such an elevated reading can induce psychological distress, including anxiety or discouragement, potentially leading to avoidance of further measurements. This psychological struggle likely occurred regardless of antihypertensive medication use. Paradoxically, interventions targeting high-risk groups with elevated blood pressure could enhance the overall blood pressure control outcomes, and our predictive model could contribute to developing high-risk strategies. Descriptive statistics in Supplementary Fig. 4 further corroborated this finding, showing that users with either higher or lower maximum SBP experienced greater dropout rates compared to those with intermediate SBP levels. It might be inferred from this observation that users within the intermediate SBP range—having not yet reached the commonly recommended target of around 135 mmHg—may experience a sense of progress and self-efficacy toward achieving that goal, which could foster greater motivation to continue monitoring and thereby lead to longer persistence. The SHAP analysis then quantified the strength and direction of this association between maximum SBP and dropout risk within our time‑series prediction model. Results demonstrated that the model can both validate and deepen our understanding of clinically relevant predictors.

Moreover, the SHAP value rankings revealed that the number of measurement days within a 2-week period was the most influential feature. Four of the top five top-ranked features (and 7 of the top 10 features) were related to measurement conditions, significantly contributing to prediction accuracy. These findings indicate that integrating both features related to measurement conditions and traditionally recognized factors such as demographic attributes [14] (e.g., sex and age) are important in predicting measurement dropout.

Regarding demographic attributes, the SHAP analysis indicated that age and sex also contributed to the predictions, with younger and older individuals and females being more likely to drop out. A possible explanation for the higher dropout rate among women is that it may relate to the unique time constraints associated with managing multiple life roles, including professional, household, and caregiving responsibilities. These time demands could make it particularly difficult to establish the consistent routines required for self-monitoring. Different factors may contribute to the observed U-shaped age trend. Among younger participants, the higher dropout risk may be due to a lower perceived urgency around hypertension, which can result in reduced motivation to maintain regular monitoring. Additionally, their more dynamic or unpredictable daily routines may hinder the formation of consistent measurement habits. In contrast, for older individuals, dropout may be influenced by age-related challenges such as cognitive decline, physical limitations that make self-measurement more difficult, or limited familiarity with app-connected digital devices. These findings are consistent with those of previous studies. For instance, Pratap et al. reported similar factors associated with continuity [14]. Descriptive statistics in the previous section also confirmed that dropout rates were higher among younger individuals and females than among the counterparts. When comparing the distribution of features between younger females and other age and sex groups, we found that younger females tended to have lower mean blood pressure than the other groups (Supplementary Table 2). This group may not have been measuring their blood pressure for managing chronic hypertension; their motivation to measure blood pressure may differ from that of the other groups. This notion could correlate with a higher likelihood of dropout. Additional survey investigations are needed to identify the specific motivations behind the measurements.

Furthermore, users who did not specify their sex during application registration were more likely to drop out both in the descriptive statistics analysis and in the SHAP-based analysis of the predictive model’s factors. While the underlying reasons for this choice are undoubtedly complex, ranging from privacy concerns to personal identity or initial engagement levels, this finding suggests that the act of registration itself contains early behavioral markers predictive of future adherence.

Consistent with these findings, the SHAP analysis revealed that changes in the number of measurement opportunities—calculated as the difference between weeks 1 and 2 within a 2-week period—were significant predictors of dropout. Among these predictors, the weekday-specific feature ranked the highest. Therefore, weekday patterns may be especially predictive of future dropout. To statistically validate these findings, we conducted additional analyses (Supplementary Fig. 5), which confirmed that changes in measurement opportunities significantly differed between the continuation and dropout groups across all time windows (entire week, weekdays, and weekends).

The stronger predictive power of weekday patterns, as identified in the SHAP analysis, may be attributed to the fact that weekday measurement behaviors have a higher temporal resolution and are less influenced by nonroutine lifestyle variability compared with weekend measurement behaviors. Moreover, isolating weekday data excludes confounding weekend-specific factors; thus, the weekday metric may be a more sensitive indicator of habit formation or decline.

### Strengths

Recently, the clinical effectiveness of home monitoring has been increasingly researched, as exemplified by studies on the blood pressure–lowering effects of home blood pressure measurements. However, studies specifically addressing interventions designed to enhance measurement engagement remain limited.

For the first time, we have demonstrated that training our model on measurement patterns can accurately predict dropout. Notably, this model can predict relatively distant future dropouts using only 2 weeks of data, allowing healthcare providers to provide interventions early to prevent their patients from discontinuing measurement. Furthermore, a key strength of our study lies in the use of large-scale real-world data from blood pressure monitor users to identify factors contributing to measurement continuation/discontinuation and to validate a generalized predictive model that demonstrates the relationship between vital sign measurement patterns and measurement continuity.

Patient support through communication tools [21] and interventions by healthcare professionals [22] effectively enhance adherence. By leveraging the predictive model developed in this study, more effective and efficient intervention strategies can be developed for rapidly expanding [4, 23–25] digital healthcare services, such as Digital Therapeutics (DTx) and RPM, to prevent dropout. Further research is required to determine the optimal timing and methods of intervention, including the content of the messages [26].

### Limitations

Although the strengths mentioned in the Strengths section are notable, the data used in this study were limited to people in Japan. Furthermore, the Omron Connect application dataset does not contain information on treatment status or medical institution involvement because of privacy and data limitations. Thus, differences in cultural backgrounds, economic conditions, and health awareness that may vary across countries and regions may have been introduced in the study. Another limitation is that the definition of “dropout” (lack of measurement for 28 consecutive days) strictly applies only to nonuse of the platform employed in this study and that it does not distinguish whether users completely stopped performing the measurements. Therefore, some users who dropped out may have continued measurements outside the platform, although the likelihood is low.

Moreover, this study focused solely on blood pressure data. The findings may be applied to other types of measurements, such as weight and blood glucose monitoring, to also support continuity.

While the presence and absence of push notifications showed no significant difference, the impact of more intensive interventions on prediction accuracy was not examined. Future research should develop intervention methods that are tailored to specific measurement patterns and evaluate their effectiveness and influence on the predictive model.

### Perspectives

Considering the limitations of this study, the generalization performance of the model using data from other countries should be evaluated to examine differences by race or region. Furthermore, the relationship between measurement patterns and continuity may vary depending on the patients’ medical history, warranting further investigation. Moreover, although this model predicts future dropout probabilities, the actual effectiveness of interventions based on these predictions has not been tested. Of note, interventions may not always be highly effective for users with high dropout probability [27]. Future research should focus on classifying users according to surveys or similar methods and optimizing intervention messages and timing accordingly [21].

The factors contributing to decreased adherence are diverse [28, 29]; however, this study did not identify specific factors such as declines in patient motivation and time constraints. In the future, factors contributing to decreased adherence should be analyzed together with predictive modeling to develop more targeted intervention strategies.

In conclusion, we developed and validated a LightGBM model to predict future discontinuation of home blood pressure measurements using only 2 weeks of vital sign and demographic data from ~295,758 Omron Connect users. The model achieved high predictive accuracy (ROC-AUC of 0.930 for 28-day and 0.851 for 56-day horizons) and identified key predictors—particularly measurement frequency patterns, maximum systolic blood pressure, and weekday-specific changes. These insights enable healthcare providers to implement timely, targeted interventions aimed at improving patient adherence to home monitoring and ultimately enhancing blood pressure control in clinical practice.

## Supplementary information

Supplementary Information
