# Supplementary material for: Predicting measurement continuity in home blood pressure monitoring using machine learning
Source: Hypertens Res. 2025 Nov 7;49(3):852–62. doi: 10.1038/s41440-025-02444-0 (PMC12960215; doi:10.1038/s41440-025-02444-0)
Supplement: Supplementary file 1 — Supplementary Information [file 41440_2025_2444_MOESM1_ESM.docx]

**Supplementary Information**

Supplemental to:

**Predicting Measurement Continuity in Home Blood Pressure Monitoring Using Machine Learning**

**Supplementary Table 1. List of features**

| Feature No. | Explanation of the features |
| --- | --- |
| 1 | Age |
| 2 | Flag indicating whether the user is female |
| 3 | Flag indicating whether sex was not provided |
| 4 | Autocorrelation coefficient of the measurement intervals |
| 5 | Mean systolic blood pressure during the first week after the initial measurement  (0: <115 mmHg, 1: 115 mmHg to <125 mmHg, 2: 125 mmHg to <135 mmHg, 3: 135 mmHg to <145 mmHg, 4: 145 mmHg to <160 mmHg, 5: ≥160 mmHg) |
| 6 | Mean diastolic blood pressure during the first week after the initial measurement  (0: <75 mmHg, 1: 75 mmHg to <85 mmHg, 2: 85 mmHg to <90 mmHg, 3: 90 mmHg to <100 mmHg, 4: ≥100 mmHg) |
| 7 | Mean blood pressure level during the first week after the initial measurement  (0: normal blood pressure, 1: high-normal blood pressure, 2: elevated blood pressure, 3: stage 1 hypertension, 4: stage 2 hypertension, 5: stage 3 hypertension) based on the Japanese Hypertension Guidelines |
| 8 | Mean pulse rate during the first week after the initial measurement (quantile) |
| 9 | Change in the number of measurement opportunities in the last 2 weeks |
| 10 | Change in weekday measurement opportunities in the last 2 weeks |
| 11 | Change in weekend measurement opportunities in the last 2 weeks |
| 12 | Change in the rate of systolic blood pressure over the last 2 weeks |
| 13 | Change in the rate of diastolic blood pressure over the last 2 weeks |
| 14 | Change in pulse rate over the last 2 weeks |
| 15 | Maximum systolic blood pressure in the last 2 weeks |
| 16 | Maximum diastolic blood pressure in the last 2 weeks |
| 17 | Maximum pulse rate in the last 2 weeks |
| 18 | Maximum interval between measurements in the last 2 weeks |
| 19 | Mean diastolic blood pressure in the last 2 weeks |
| 20 | Maximum diastolic blood pressure in the last 2 weeks |
| 21 | Mean pulse rate in the last 2 weeks |
| 22 | Mean measurement interval in the last 2 weeks |
| 23 | Variability in measurement frequency in the morning over the last 2 weeks |
| 24 | Variability in measurement frequency in the afternoon over the last 2 weeks |
| 25 | Variability in the time of the first measurement each day over the last 2 weeks |
| 26 | Minimum systolic blood pressure in the last 2 weeks |
| 27 | Minimum diastolic blood pressure in the last 2 weeks |
| 28 | Minimum pulse rate in the last 2 weeks |
| 29 | Minimum interval between measurements in the last 2 weeks |
| 30 | Number of days since the last measurement |
| 31 | Number of measurements in the last 2 weeks |
| 32 | Number of measurement days in the last 2 weeks |
| 33 | Mean number of measurements per opportunity |
| 34 | Number of morning (3:00–11:59) measurements in the last 2 weeks |
| 35 | Number of afternoon (12:00–17:59) measurements in the last 2 weeks |
| 36 | Number of evening (18:00–26:59) measurements in the last 2 weeks |
| 37 | Number of weekday measurements in the last 2 weeks |
| 38 | Number of weekend measurements in the last 2 weeks |
| 39 | Number of Monday measurements in the last 2 weeks |
| 40 | Number of Tuesday measurements in the last 2 weeks |
| 41 | Number of Wednesday measurements in the last 2 weeks |
| 42 | Number of Thursday measurements in the last 2 weeks |
| 43 | Number of Friday measurements in the last 2 weeks |
| 44 | Number of Saturday measurements in the last 2 weeks |
| 45 | Number of Sunday measurements in the last 2 weeks |
| 46 | Number of measurement opportunities in the last 2 weeks |
| 47 | Number of morning (3:00–11:59) measurement opportunities in the last 2 weeks |
| 48 | Number of afternoon (12:00–17:59) measurement opportunities in the last 2 weeks |
| 49 | Number of evening (18:00–26:59) measurement opportunities in the last 2 weeks |
| 50 | Number of weekday measurement opportunities in the last 2 weeks |
| 51 | Number of weekend measurement opportunities in the last 2 weeks |
| 52 | Number of Monday measurement opportunities in the last 2 weeks |
| 53 | Number of Tuesday measurement opportunities in the last 2 weeks |
| 54 | Number of Wednesday measurement opportunities in the last 2 weeks |
| 55 | Number of Thursday measurement opportunities in the last 2 weeks |
| 56 | Number of Friday measurement opportunities in the last 2 weeks |
| 57 | Number of Saturday measurement opportunities in the last 2 weeks |
| 58 | Number of Sunday measurement opportunities in the last 2 weeks |
| 59 | Number of measurement days in the first week after the initial measurement (quantile) |
| 60 | Number of measurement days in the first 2 weeks after the initial measurement (quantile) |
| 61 | Time interval between the initial measurement and the second measurement (quantile) |
| 62 | Number of days since the initial measurement |
| 63 | Change in systolic blood pressure relative to the mean systolic blood pressure during the first week after the initial measurement (quantile) |
| 64 | Change in diastolic blood pressure relative to the mean diastolic blood pressure during the first week after the initial measurement (quantile) |
| 65 | Change in the pulse rate relative to the mean pulse rate during the first week after the initial measurement (quantile) |
| 66 | Variance in systolic blood pressure over the last 2 weeks |
| 67 | Variance in diastolic blood pressure over the last 2 weeks |
| 68 | Variance in pulse rate over the last 2 weeks |

**Supplementary Table 2. Feature distributions were compared across four groups: females aged <40 years (Group 1), nonfemales aged <40 years (Group 2), females aged ≥40 years (Group 3), nonfemales aged ≥40 years (Group 4).**

| Feature | p-value  (Group 1  vs.  Group 2) | p-value  (Group 1  vs.  Group 3) | p-value  (Group 2  vs.  Group 4) | p-value  (Group 3  vs.  Group 4) | Group 1_mean | Group 1_std | Group 2_mean | Group 2_std | Group 3_mean | Group 3_std | Group 4_mean | Group 4_std |
| --- | --- | --- | --- | --- | --- | --- | --- | --- | --- | --- | --- | --- |
| The 2-week mean SBP | 0.0 | 9.36E-189 | 0.13 | 0.0 | 118.46 | 14.47 | 128.33 | 13.78 | 124.20 | 13.30 | 128.53 | 12.65 |
| The 2-week mean DBP | 6.07E-98 | 1.57E-14 | 8.61E-4 | 0.0 | 79.83 | 11.76 | 83.65 | 10.99 | 81.01 | 9.31 | 84.00 | 9.47 |
| The 2-week mean pulse | 2.75E-27 | 0.0 | 3.64E-35 | 0.0 | 74.98 | 9.17 | 73.34 | 10.60 | 70.12 | 8.94 | 72.12 | 10.28 |
| SBP level at the time of entry | 1.57E-195 | 1.52E-97 | 2.73E-35 | 0.0 | 1.41 | 1.44 | 2.09 | 1.37 | 1.81 | 1.38 | 2.24 | 1.31 |


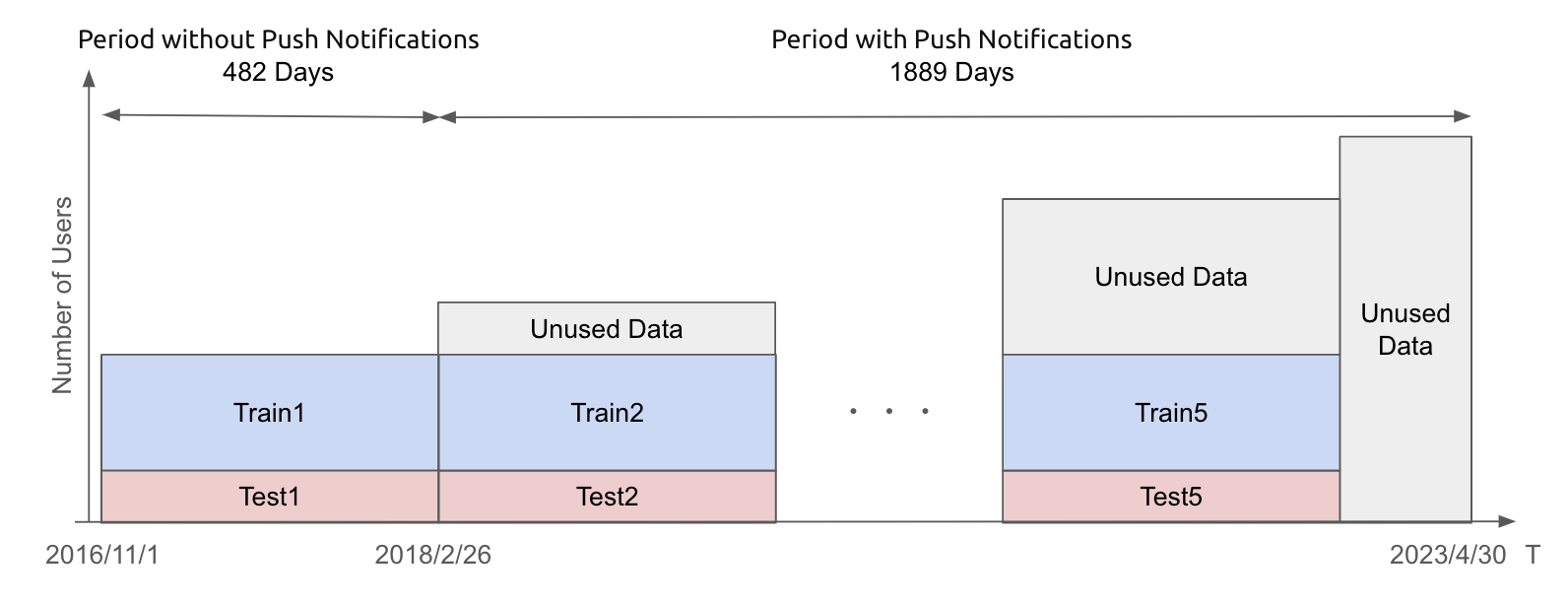


**Supplementary Figure 1. Training/test data–splitting method：Comparison of Prediction Accuracy With and Without Push Notifications**

As mentioned in Section 3.1, to determine whether push notifications affected the prediction accuracy of the model, we evaluated data from February 26, 2018, onwards when some users received push notifications. For the training and validation of the model during the period without push notifications, we used data from 80% of the users who performed measurements between November 1, 2016, and February 26, 2018. The data of the remaining 20% of the users served as the test data (Test 1). For the period with push notifications, we divided the data into subsets of 482 days and prepared four training and validation datasets (Train 2 to Train 5) and the corresponding test datasets (Test 2 to Test 5) to evaluate the model’s generalization performance. The 482-day period segmentation was made to match the notification-free period between November 1, 2016, and February 26, 2018. Furthermore, to ensure comparability, we ensured that the number of users in both the training/validation and test datasets during the push notification period was the same as that during the non–push notification period. All measurement data from the users included in the training/validation were excluded from the accuracy evaluation datasets.


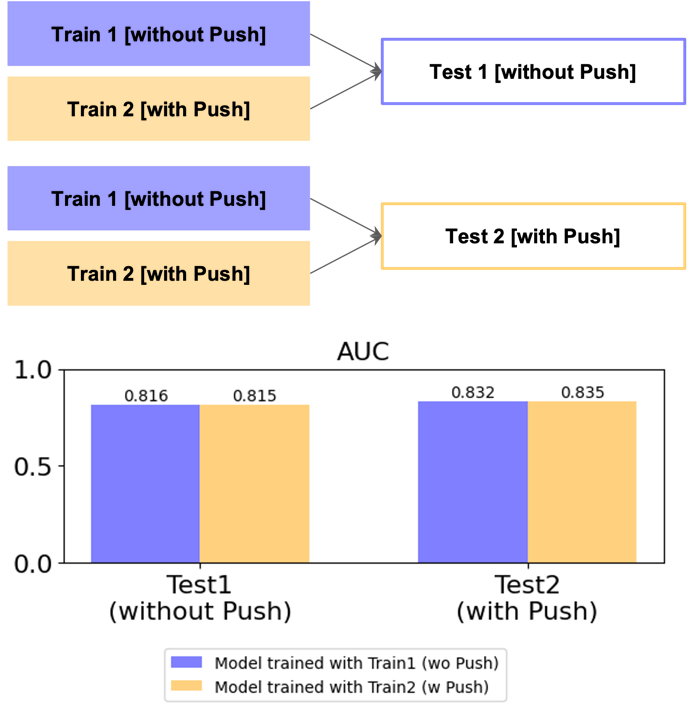


**Supplementary Figure 2. Comparison of the evaluation method and accuracy between periods with and without push notifications (M = 56)**

We used the prepared datasets described in Supplementary Figure 1 to compare model accuracy between models trained on data from the push notification period (Train 2 to Train 5) and those trained on data from the non–push notification period (Train 1). Supplementary Figure 2. summarizes the evaluation method and the results. Given that the accuracy of Train k (k = 3,4,5) and Test k (k = 3,4,5) was comparable to those using Train 2 and Test 2, we present only the results for Train 1, Train 2, Test 1, and Test 2.

From the results, the ROC-AUC remained consistent between the models trained on data from the push notification periods and those trained on data from non–push notification periods, regardless of the presence of push notifications in the test data. Thus, the presence or absence of push notifications during the training data period may have not affected the model’s accuracy.

Importantly, this analysis evaluated the effect of push notifications on the prediction accuracy of the machine learning model, not on the blood pressure measurement behavior itself. In other words, the consistency of model performance across both periods suggests that the model successfully learned robust behavioral patterns regardless of whether users had received push notifications or not. However, this result does not imply that push notifications had no impact on users’ actual adherence to measurement. Rather, the model likely captured intervention-induced behavioral changes, such as increased or decreased measurement frequency, and incorporated them into its prediction process. Therefore, our analyses should not be interpreted as a clinical evaluation of the effectiveness of push notifications on patient behavior.


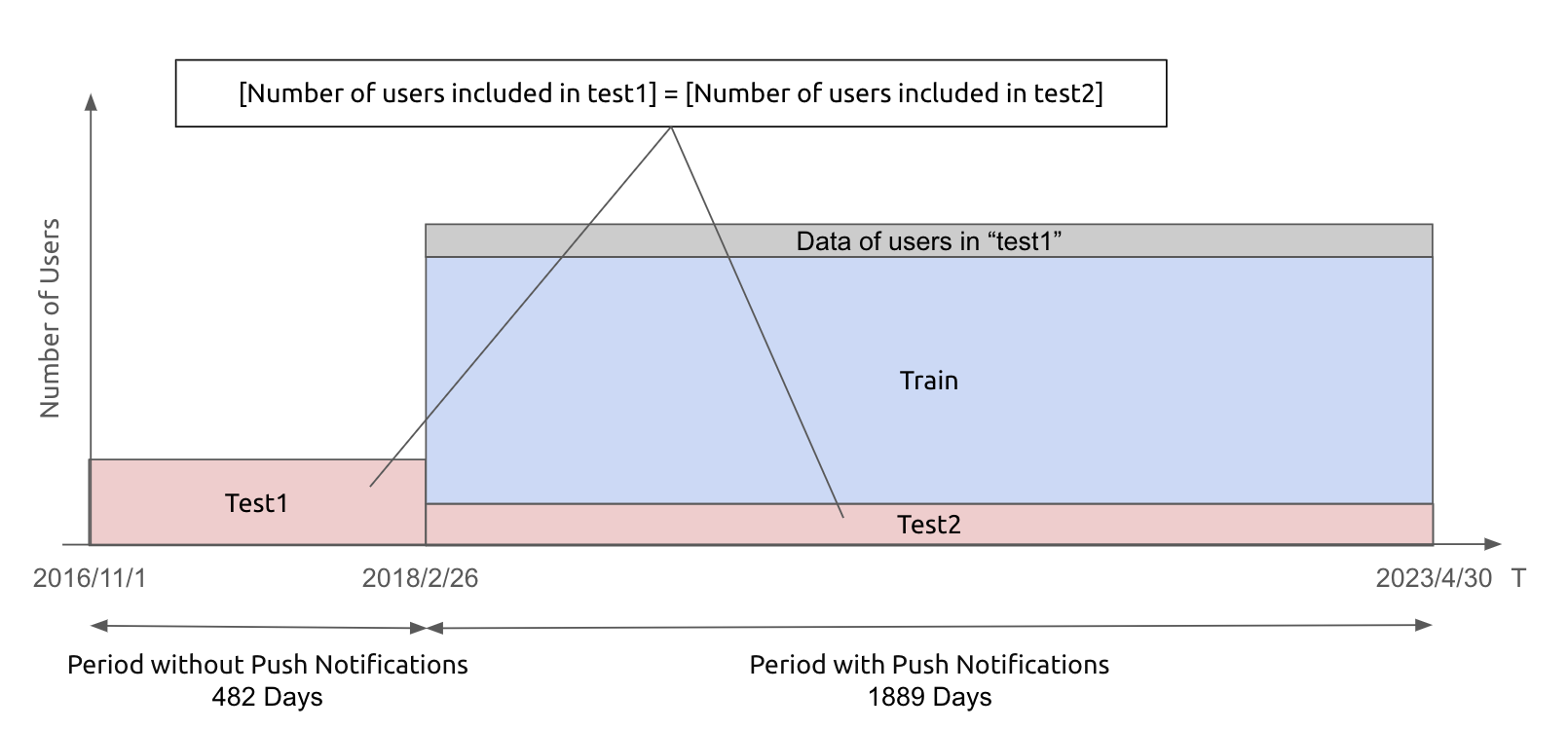


**Supplementary Figure 3. Training/test data–splitting method: Method of splitting the training/test data used for model validation**


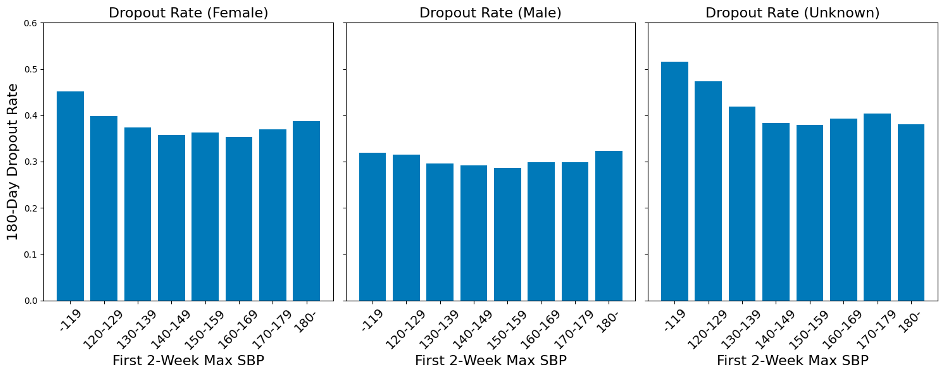


**Supplementary Figure 4. 180-day dropout rate by maximum SBP stratified by sex**

We investigated the relationship between blood pressure readings and discontinuation risk over 180 days for each sex cohort. For every user, we determined the maximum systolic blood pressure (SBP) recorded during the initial 14-day monitoring window. We then grouped the users by sex (female, male, and unknown). Subsequently, in each group, we calculated the proportion of individuals who stopped measuring within 180 days for each SBP category.

Both female and male cohorts exhibited a pronounced U-shaped association. Dropout rates were higher in the lowest and highest maximum-SBP categories than in midrange categories. The unknown-sex group followed a broadly similar pattern, although its highest SBP category (≥180 mmHg) showed a relative decrease in dropout, partially attenuating the U-shaped curve. Thus, extreme maximum SBP values early in the monitoring period may be linked to the subsequent discontinuation risk across sex groups.


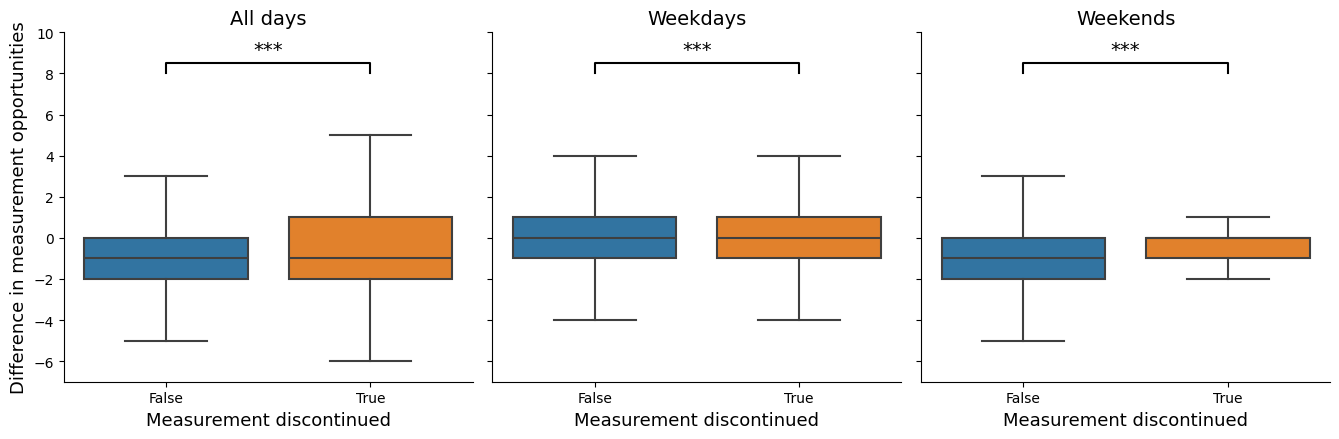


**Supplementary Figure 5. Group differences in changes in measurement opportunities between participants who continued measurement and those who did not**

To examine day-specific differences in measurement behavior, we compared the changes in measurement opportunities between participants who continued home monitoring and those who did not. Such changes were calculated as the difference in the number of measurement days between weeks 1 and 2 of a 2-week observation period. Changes in measurement opportunities significantly differed between the two groups across all days, weekdays, and weekends (p < 0.001, unpaired t-test).
